# The secreted FolAsp aspartic protease facilitates the virulence of *Fusarium oxysporum* f. sp. *lycopersici*

**DOI:** 10.3389/fmicb.2023.1103418

**Published:** 2023-01-25

**Authors:** Chenyang Wang, Yaning Zheng, Zhishan Liu, Yongpan Qian, Yue Li, Limei Yang, Sihui Liu, Wenxing Liang, Jingtao Li

**Affiliations:** ^1^College of Plant Health and Medicine, Engineering Research Center for Precision Pest Management for Fruits and Vegetables of Qingdao, Shandong Engineering Research Center for Environment-Friendly Agricultural Pest Management, Shandong Province Key Laboratory of Applied Mycology, Qingdao Agricultural University, Qingdao, China; ^2^College of Science and Information, Qingdao Agricultural University, Qingdao, China

**Keywords:** *Fusarium oxysporum*, effector, virulence, plant defense, protease activity

## Abstract

Pathogens utilize secretory effectors to manipulate plant defense. *Fusarium oxysporum* f. sp. *lycopersici* (*Fol*) is the causal agent of *Fusarium* wilt disease in tomatoes. We previously identified 32 secreted effector candidates by LC-MS analysis. In this study, we functionally identified one of the secreted proteins, FolAsp, which belongs to the aspartic proteases (Asp) family. The FolAsp was upregulated with host root specifically induction. Its N-terminal 1–19 amino acids performed the secretion activity in the yeast system, which supported its secretion in *Fol*. Phenotypically, the growth and conidia production of the *FolAsp* deletion mutants were not changed; however, the mutants displayed significantly reduced virulence to the host tomato. Further study revealed the FolAsp was localized at the apoplast and inhibited INF1-induced cell death *in planta*. Meanwhile, FolAsp could inhibit flg22-mediated ROS burst. Furthermore, FolAsp displayed protease activity on host protein, and overexpression of FolAsp in *Fol* enhanced pathogen virulence. These results considerably extend our understanding of pathogens utilizing secreted protease to inhibit plant defense and promote its virulence, which provides potential applications for tomato improvement against disease as the new drug target.

## Introduction

Plants are challenged by a wide variety of microbes and parasites. Thanks to their multilayered physical barriers, preformed defenses, and innate immune system, plants can defeat most potential microbial invaders (Zhang et al., 2017; Cao et al., 2018). However, pathogens can secrete abundant proteins to suppress plants’ innate immune in order to effectively infect the hosts (Gawehns et al., 2015). These proteins are mostly secreted extracellularly *via* the endoplasmic reticulum (ER)/Golgi pathway, which depends on their N-terminal signal peptides. These protein functions range from altering plant cellular metabolic pathways and signaling cascades, RNA silencing, antimicrobial inhibition, and interfering with recognition machinery (Sharpee and Dean, 2016). The importance of understanding the function of secreted proteins has given rise to the field of pathogen–host interaction.

Proteins secreted from pathogens can be divided into two classes according to their functions: enzymatic functional proteins and non-enzymatic functional proteins (Qian et al., 2022b). Cell wall-degrading enzymes (CWDEs), cutinases, proteases, peptidases, and ribonucleases can be secreted from pathogens to proceed with enzymatic function. For instance, the glycoside hydrolase family 12 (GH12) protein, XEG1, produced by the soybean pathogen *Phytophthora sojae* exhibits xyloglucanase and beta-glucanase activity (Ma et al., 2015). In addition, another two GH12 proteins, FoEG1 and VdEG1, secreted by *Fusarium oxysporum* and *Verticillium dahliae*, respectively, are essential for fungal virulence *via* its cellulase activity (Gui et al., 2017; Zhang L. et al., 2021). In *Botrytis cinerea*, Xyn11A, xylanase, is required for virulence in host plants (Noda et al., 2010). Another class is composed of many low-molecular-weight proteins, which are rich in cysteine and lack known enzyme activity (Hacquard et al., 2012; Lyu et al., 2016). Some of these secreted proteins are among a repertoire of pathogenicity effectors.

The aspartic protease was first isolated from *Aspergillus niger*, named aspergillopepsin I (EC 3.4.23.18), which is a typical pepsin aspartic protease active between pH 2 and 4 and is inhibited by pepstatin (Song et al., 2020). Some of the aspergillopepsin-like family proteins from various fungal species may serve as virulence factors beyond its wide range of commercial applications as industrial aids (Song et al., 2020). For example, the aspergillopepsin-like proteins (cd06097) contribute to virulence in the human pathogen *Candida* (Monika and Zbigniew, 2017). A type III-independent extracellular aspartic protease Rsa1e is identified as a virulence factor of *Ralstonia solanacearum* in potatoes (Jeong et al., 2011). Aspergillopepsin from *Aspergillus fumigatus* is involved in invasive aspergillosis owing to its elastolytic activity.

*Fusarium oxysporum* f. sp. *lycopersici* (*Fol*), the causal agent of *Fusarium* wilt limited to tomato, invades the roots and subsequently colonizes the xylem vessels, thereby compromising water transport resulting in wilting (Michielse and Rep, 2009). During the colonization of the host xylem vessels, *Fol* secretes small effectors, including 14 different “Secreted-in-Xylem” (Six) proteins, and some of them play critical roles in determining plant immunity (Houterman et al., 2007; Lievens et al., 2009; Ma et al., 2013; Gawehns et al., 2015). In our previous studies, we have identified 32 secreted effector candidates in *Fol* by mass spectrometry, which gives the first clues into the establishment of *Fol* early infection (Li J. et al., 2020). To date, the FolSvp1 is characterized to contribute virulence by translocating a key defense protein PR1 to dampen the host defense (Li et al., 2022). FoRnt2, a secreted ribonuclease T2 protein, contributes to the full virulence of *F. oxysporum* in tomato plants depending on its RNase activity (Qian et al., 2022a). The FoAYP1 contributes to the virulence of *Fol* through peptidase activity on host protein (Qian et al., 2022b). However, the function of some other secreted proteins in fungal virulence remains largely unknown.

In this study, we identified one *Fol*-secreted aspartic protease FolAsp, which belongs to the aspergillopepsin-like protein family. FolAsp was found to contribute to the full virulence of *Fol* in tomato seedlings and exhibited protease activity to host roots. FolAsp was located at the apoplast *in planta*, which displayed the inhibition activity of plant ROS burst. These findings provide another example in which the apoplast effector contributed virulence *via* its protease activity, directly facilitating the successful outcome of a fungal plant pathogen invasion.

## Materials and methods

### Plants, fungal strains, and cultural conditions

The WT tomato (*Solanum lycopersicum* cv. Alisa Craig [AC]) was used and potted in a soil mix (vermiculite:humus = 1:2) and placed in a climatized greenhouse at 28°C with 65% relative humidity and 16-h light/8-h dark cycle at 25°C (Cao et al., 2018). The *Fol* strain Fol4287 (Li J. et al., 2020) was used and cultured on potato dextrose agar (PDA) plates at 25°C. To harvest conidia, mycelial plugs were inoculated in 150 ml of potato dextrose broth (PDB) liquid medium with shaking at 180 rpm at 25°C overnight.

### RNA extraction and qRT-PCR assay

For qRT-PCR of *FolAsp* in response to root induction, the harvested *Fol* conidia (10^8^ conidia) were suspended in 100 ml of 10% liquid YPD medium (1% yeast extract, 2% peptone, and 2% dextrose) amended with 50 mg aseptic tomato root or not, and cultured at 25°C with shaking at 150 rpm. The aseptic tomato seedlings were grown in half-strength MS media (1/2 MS). Then, *Fol* cultures at different time points (0, 3, 6, 9, and 12) were harvested by pelleting the developed conidia in the centrifuge at 10,000 rpm for 5 min and finally ground in liquid nitrogen. Total RNA was isolated using Trizol reagent (Invitrogen) according to the manufacturer’s instructions. Total RNA (2 μg) was used for reverse transcription with the PrimeScript™ RT reagent Kit (TaKaRa). Quantitative expression assays were performed by using the 2x M5 HiPer SYBR Premix EsTag kit (Mei5bio) and a LightCycle^®^96 (Roche) real-time PCR detection system. The relative quantification of gene expression was performed using the 2^–ΔΔct^ method (Li J. et al., 2018). RNA samples were extracted from three biological replicates. Data were normalized against the *Fol* histone H4-specific gene *FolH4*. The primer pairs used for real-time PCR are listed in Supplementary Table 1.

### Constructs for gene deletion, complementation, and overexpression in *Fol*

To generate a *FolAsp* gene deletion mutant, two 0.7-kb fragments flanking the target gene were PCR amplified with the primer pairs FolAsp-up-F/R and FolAsp-down-F/R. The 5’ and 3’ flanking sequences of the target gene and the hygromycin resistance gene cassette (*Hph*) were amplified with primer pair Hph-F/R by overlap PCR. The resulting PCR fragment was purified for protoplast transformation of *Fol*. To construct the complementation vectors, a genomic region encompassing the entire *FolAsp* coding sequence and its native promoter region was amplified using the primers FolAsp-C-F/R and inserted into the pYF11 (G418 resistance) vector using the ClonExpress II One Step Cloning Kit (Vazyme). The resulting vectors were transformed into the corresponding knockout mutants Δ*FolAsp*-KO11 by protoplast transformation. To overexpress FolAsp1-GFP, we amplified *FolAsp* using the primers FolAsp(OE)-F and FolAsp-C-R and inserted them into the *Xho*I-digested pYF11 vector carrying the RP27 promoter. Then the construct was used for the protoplast transformation of WT *Fol*. All the primers used in this study are listed in Supplementary Table 1.

### Functional verification of FolAsp signal peptide

The predicted N-terminal 19-amino-acid signal peptide sequences of FolAsp were fused in frame to the invertase gene in the pSUC2 vector by two complementary primer sequences (Supplementary Table 1). *Eco*RI and *Xho*I restriction enzymes were used to insert the signal peptide sequences into the pSUC2 vector (Yin et al., 2018). The recombinant plasmids were then transformed into invertase secretion-deficient yeast strain YTK12 by lithium acetate-mediated transformation. The function of the signal peptides can be evaluated by using different selective media and color reactions to verify the secretion of invertase as described previously (Yin et al., 2018).

### Construction of binary vectors and transient expression

For the subcellular localization of FolAsp *in planta*, the full coding sequence of *FolAsp* was PCR amplified using primer pairs FolAsp-pQB-F/R. The forward primer FolAsp1^SlPR1SP^-PQB-F was used to generate an artificial form of *FolAsp* containing the tomato SlPR1 signal peptide (1–24 amino acids) instead of its endogenous signal peptide (1–19 amino acids) (Li et al., 2022). These constructs were cloned into a pQB-V3 vector (Chen et al., 2020) and then transferred to the C-terminal GFP-tagged pK7FWG2 (Karimi et al., 2002) using Gateway LR Clonase Enzyme Mix (Invitrogen). Later, these binary constructs were transformed into *Agrobacterium* LBA4404 for transient expression in *N. benthamiana* as described previously (Li H. et al., 2018). The oomycete PAMP INF1-expressed INF1 *Agrobacterium* was used as a positive control to trigger cell death (Qiu et al., 2022). A strain containing the silencing suppressor P19 was co-infiltrated at an OD600 of 0.3 for enhancing the expression of the target genes. For plasmolysis, leaf tissues were cut and incubated for 6 h with 800 mM mannitol and then imaged under the Olympus BX53 fluorescence microscope (Melville, NY, USA).

### Protein extraction and western blotting

The plant tissues were frozen and ground in liquid nitrogen and then homogenized in a protein extraction buffer and a protease inhibitor mixture (Solarbio) as described previously (Xian et al., 2020). The obtained proteins were separated by 12% SDS-PAGE gel and immunoblotted using anti-GFP (1:5000 dilution, Abcam, ab183734). The signals in the blots were visualized with ECL kits (Millipore) and photographed using an Amersham Imager 680 (GE Healthcare). SDS-PAGE was performed with Coomassie brilliant blue (CBB) straining to verify equal loading.

### Prokaryotic expression and purification of FolAsp

*FolAsp* sequences lacking the N-terminal signal peptide were cloned into the pET-28a expression vector *via* the overhangs added by the primers during amplification (Supplementary Table 1). The His-FolAsp fusion proteins were expressed in *E. coli* BL21 cells as described previously (Gamir et al., 2016). Briefly, isopropyl β-D-thiogalactoside (IPTG) was added into the medium to induce target protein expression and then cultured at 25°C and 180 rpm for 16 h. The harvested cells were resuspended in lysis buffer containing 20 mM Tris-HCl, 500 mM NaCl, 20 mM imidazole, and 1 mM phenylmethylsulfonyl fluoride (PMSF) and lysed with a microfluidizer. The proteins were purified using Ni-nitrilotriacetic acid (Ni-NTA) affinity resin (Qiagen) following the manufacturer’s instructions. The eluted fraction was desalted on a Sephadex G-25 column (PD-10, GE Healthcare) equilibrated with 50 mM Tris-HCl (pH 8). The protein concentrations were determined with an Omni-Easy Instant BCA Protein Assay Kit (Epizyme). The purified protein was concentrated with a 10-kDa MWCO Amicon Ultra Centrifugal Filter (Merck Millipore) and visualized by Coomassie-stained SDS-PAGE.

### Protease activity assay

To determine whether FolAsp could degrade host protein, root protein was extracted and inoculated with purified FolAsp or a spore suspension (5 × 10^6^ spores/ml) of the corresponding *Fol* strains for 2 or 4 h, respectively. The treated root protein was subsequently used for SDS-PAGE and CBB staining to evaluate the protein change induced by FolAsp.

### Disease assays

Pathogenicity assays of *Fol* were performed using the modified root dip method (Di et al., 2016; Zhang N. et al., 2021). The conidia at the optimum concentration of 5 × 10^6^ conidia/ml (Vitale et al., 2019) were used for the infection assay and antifungal assay. Briefly, 14-day-old tomato seedlings were uprooted from the soil, inoculated with a spore suspension (5 × 10^6^ spores/ml) of *Fol* for 10 min, and subsequently potted and kept at 25°C. Two weeks later, disease symptoms of plants were scored using a disease index ranging from 0 to 5 (0, no symptoms; 1, 0–20% defoliated leaves; 2, 20–40% defoliated leaves; 3, 40–60% defoliated leaves; 4, 60–80% defoliated leaves; and 5, 80–100% defoliated leaves).

### A burst of reactive oxygen species (ROS)

Reactive oxygen species production was monitored with a luminol/peroxidase-based assay as described previously (Sang and Macho, 2017). In brief, the sterile H_2_O-balanced tomato leaf discs in a 96-well white plate were replaced with the luminol (100 μM/L)/peroxidase (20 μg/ml) reaction solution supplied with sterile water or 50 nM flg22 (Genscript Biotech Corporation, China). Luminescence was measured using the Multimode Microplate Reader (Varioskan LUX, Thermo Scientific, USA).

### DAB staining and H_2_O_2_ quantification

Detection of hydrogen peroxide was conducted using 3,3-diaminobenzidine (DAB) from Sigma-Aldrich. Briefly, the detached tobacco leaves were immersed in a 1 mg/ml solution of DAB in buffer (pH 3.8) and incubated at room temperature for 8 h in the dark. Then, the leaves were bleached with 95% ethanol until the samples became colorless (Yang et al., 2022).

The content of H_2_O_2_ was analyzed with a hydrogen peroxide assay kit (Beyotime, S0038) according to the manufacturer’s protocols. In brief, 10 mg of plant tissue was ground into a homogenate with 200 μl of lysate and centrifuged at 12,000 × *g* for 5 min at 4°C. The reaction mixture containing 50 μl of supernatant and 100 μl of H_2_O_2_ detection reagent was left at room temperature (20–25°C) for 30 min and then immediately measured with a spectrometer at a wavelength of 560 nm. Absorbance values were calibrated to a standard curve generated with known concentrations of H_2_O_2_.

### Statistical analysis

All graphs were exported using the GraphPad Prism 6 software (La Jolla, CA, USA). Statistical analysis was performed by analysis of variance (ANOVA) or unpaired Student’s *t*-test methods.

## Results

### Expression profiling of *FolAsp* in response to the host root

**FIGURE 1:**
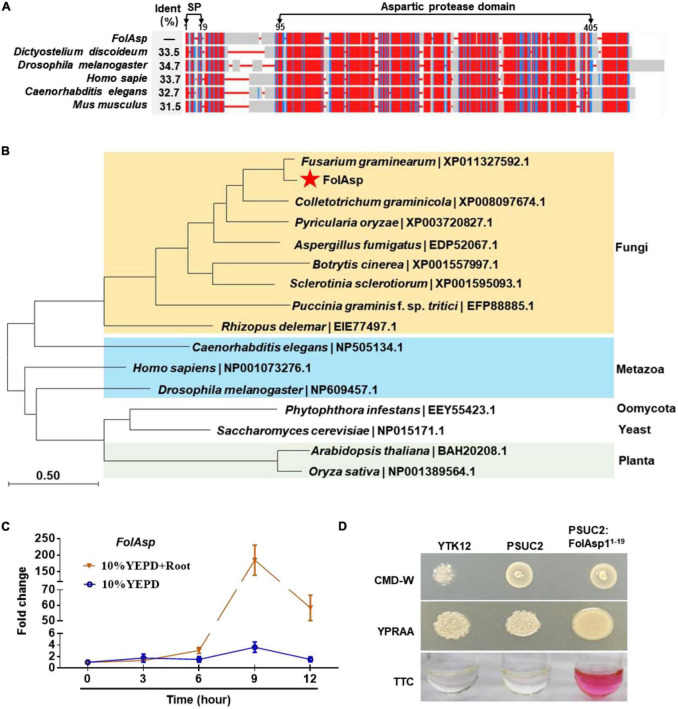
Conservation, expression, and secretion analysis of FolAsp protein. **(A)** SmartBLAST was used to locate the regions of high sequence conservation in a graphical view among different model organisms. The identity (Ident) of the FolAsp and its homologs were as indicated. The predicted signal peptide (SP) and aspartic protease domain were marked on the top of the graph. **(B)** Phylogenetic analysis of Asp proteins in *Fol* and other species. The phylogenetic tree was generated by NJ method in MEGA. **(C)** Expression profile of *FolAsp* in *Fol* with tomato root induction at the indicated times measured by qRT-PCR. The expression levels were normalized to that of the histone H4 gene *FolH4*. Data represent means ± SE of three independent replicates. **(D)** Functional validation of the signal peptide (1–19 amino acids) of FolAsp. The indicated strains were grown on CMD-W or YPRAA medium for 3 days. TTC was used to test the enzymatic activity of reducing TTC to red TPF.

From the previously identified *Fol* secretome, we found that a hypothetical protein (FOXG_03994, XP_018238471.1) consists of 408 amino acids with a signal peptide at the N-terminus, which is homologous to aspartic protease protein (ASP) from other fungal species (Li J. et al., 2020). This protein was then termed as FolAsp in *Fol*, which was predicted to contain an ASP domain at the C-terminus by SmartBLAST (Figure 1A). The SmartBLAST analysis results displayed a wide taxonomic diversity in mycetozoa (*Dictyostelium discoideum*), fruit fly (*Drosophila melanogaster*), and animal species (*Mus musculus*, *Homo sapiens*, and *Caenorhabditis elegans*). Meanwhile, phylogenetic analysis of the putative amino acid sequence of Asp homologs showed that FolAsp was much more closely related to *Fusarium* taxa and other pathogenic fungi, compared with metazoan species (Figure 1B). However, the FolAsp was far away with its homologs from plants, yeast, or oomycete, which is consistent with no conserved homologs in these species found by SmartBLAST searching. These results suggested that FolAsp was conserved in the fungi and metazoan.

Inducing with tomato root, *FolAsp* is upregulated expressing during conidia generation (Li J. et al., 2020). To more precisely define the regulation of *FolAsp* expression, we examined its transcript accumulation under different cultured conditions by quantitative reverse transcription-polymerase chain reaction (qRT-PCR). The expression profile revealed transcript accumulation of *FolAsp* was significantly higher across developmental stages in 10% YEPD medium with tomato root, compared with in 10% YEPD medium alone (Figure 1C). This indicates that the host root could specifically enhance the upregulation expression of *FolAsp* gene.

### Secretory activity analysis of FolAsp N-terminal signal peptide

FolAsp is believed to contain a predicted 19-amino-acid signal peptide at the N-terminus according to SignalP-5.0,^1^ which indicates that FolAsp is a secreted protein (Figure 1A), in accordance with its existence in the *Fol* secretome (Li et al., 2022). Here, we further performed a yeast signal trap assay to validate the secretory function of the signal peptide (Yin et al., 2018). As shown in Figure 1D, the YTK12 strain carrying pSUC2-FolAsp^1–19^ was able to grow on YPRAA medium with raffinose as the sole carbon source and reduce the triphenyltetrazolium chloride (TTC) to the red-colored insoluble triphenylformazan (TPF), indicating that the N-terminal signal peptide of FolAsp is functional for its secretion.

### FolAsp is not involved in mycelial growth or conidiation

To investigate the biological function of the FolAsp, we used the split-marker method to generate a gene replacement construct containing an *Hph* gene (Supplementary Figure 1A) and transformed the construct into protoplasts of the wild-type (WT) *Fol*. Five deletion mutants (Δ*FolAsp*-10, Δ*FolAsp*-11, Δ*FolAsp*-15, Δ*FolAsp*-28, and Δ*FolAsp*-29) were obtained on 100 mg/ml hygromycin plates. Homologous recombination events were verified by PCR (Supplementary Figure 1B), with the primers listed in Supplementary Table 1. To further confirm that the change in Δ*FolAsp* strains occurred because of *FolAsp* gene deletion, *FolAsp* gene complementation transformants were produced from Δ*FolAsp*-10 protoplasts following transformation with the full-length *FolAsp* gene including its upstream 1,000 bp sequences as the native promoter. Transformants were selected on potato dextrose agar (PDA) media with 100 mg/ml G418, and two PCR-positive transformants were purified and verified by PCR. One recovered strain Δ*FolAsp*-C (Supplementary Figure 1C) was chosen for detailed phenotypic characterization.

**FIGURE 2:**
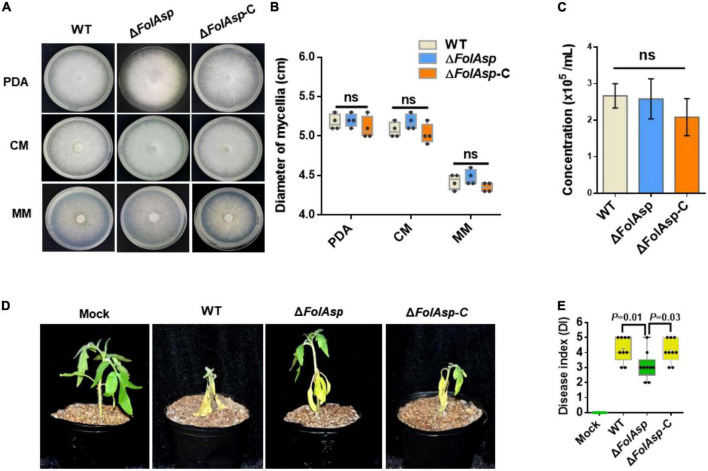
Phenotypic analysis of FolAsp mutant strains. **(A)** Mycelial growth of the WT and Δ*FolAsp* (Δ*FolAsp*-KO10) mutant strains on the indicated plates after 5 days of cultivation. **(B)** Mycelium diameters of colonies of the strains in panel **(A)** were measured at 5 days of cultivation. **(C)** Conidia production of the WT and Δ*FolAsp* mutant strains in PDB liquid medium at the indicated times. No significant difference (ns) was indicated according to multiple *t*-test. **(D)** Virulence of the wild-type (WT) and *FolAsp* mutant strains on tomato. The disease symptoms were observed, and photographs were taken at 14 days after inoculation (DAI). Mock, inoculation with water. Experiments were repeated three times with similar results. **(E)** Disease index scored at 14 DAI. Significant differences were determined according to one-way ANOVA, and *p* value was shown. For panels **(B,E)** whiskers of the boxplots display the upper and lower quartiles, the boxes display the interquartile range, and the plus displays the mean.

We first compared the growth phenotypes of the knockout strain with those of the WT and complementary (Δ*FolAsp*-C) strains to determine the potential function of FolAsp. No differences in colony morphology were found among the WT, Δ*FolAsp*, and Δ*FolAsp*-C strains on PDA media, complete media (CM), and minimal media (MM) (Figure 2A). In addition, the growth rate of the Δ*FolAsp* strains was similar to that of the WT and Δ*FolAsp*-C strains on media under the same conditions (Figure 2B). To evaluate the role of *FolAsp* in conidiation, we also inoculated mycelial plugs of all strains in carboxymethyl cellulose (CMC) liquid media for different time series. Our quantitative data showed that loss of the *FolAsp* gene did not affect the conidiation of the Δ*FolAsp* strain compared with the WT and Δ*FolAsp*-C strains (Figure 2C), suggesting that FolAsp is not required for the conidiation of *F. oxysporum*. Taken together, our results revealed that FolAsp did not affect vegetative hypha growth or conidia production.

### FolAsp contributes full virulence of *Fol*

To investigate the possible function of FolAsp in *Fol* virulence, we observed changes in symptoms of tomato seedlings during inoculation. Three-week-old seedlings were inoculated with conidia of the WT and Δ*FolAsp* strains (KO-10; 11, 15, 28, and 29) at 25°C for 14 days. Obvious disease symptoms, such as stunted plant growth and leaf yellowing, occurred for tomato seedlings inoculated with the WT strain, which showed higher infection ability, whereas significantly reduced disease symptoms were detected in tomato seedlings inoculated with all the five knockout strains (Supplementary Figure 2). Furthermore, we confirmed its virulence function on tomato seedlings by comparing WT, Δ*FolAsp*, and complementary Δ*FolAsp*-C strains. Obviously, the virulence of the knockout mutant was strongly reduced in tomato seedlings (Supplementary Figure 2) at 14 days after inoculation (DAI). However, the Δ*FolAsp*-C strain displayed a higher disease index, which was similar to WT under the same inoculation conditions (Figures 2D, E). These results indicate that *FolAsp* is required for the full virulence of *Fol*.

**FIGURE 3:**
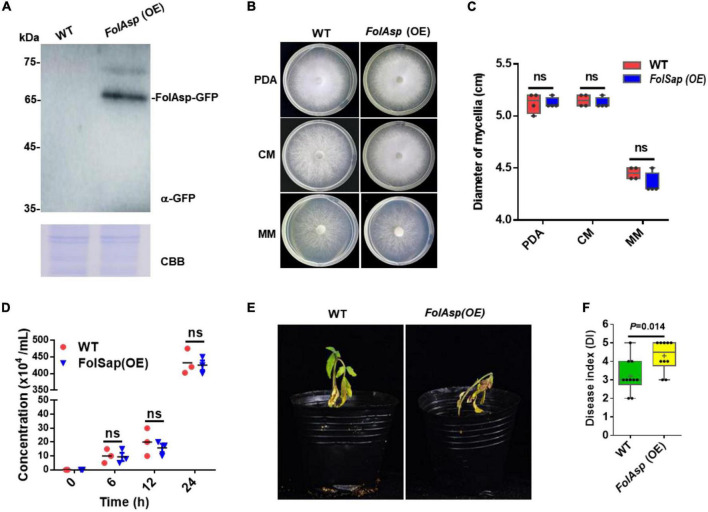
FolAsp-GFP overexpression increases virulence. **(A)** FolAsp-GFP overexpression [FolAsp (OE)] in *Fol* was determined by Western blotting with anti-GFP antibody. The WT strain was used as the control. Protein loading is indicated by CBB staining. **(B)** Mycelial growth of the WT and *FolAsp* (OE) strains on PDA plates after 5 days of cultivation. **(C)** Mycelium diameters of colonies of the strains in panel **(B)** were measured at 5 days of cultivation. **(D)** Conidia production of the WT and *FolAsp* (OE) strains in PDB liquid medium at the indicated times. No significant difference (ns) was indicated according to multiple *t*-test. **(E)** Virulence of the WT- and *FolAsp*-overexpressed mutant strains on tomato. **(F)** Disease index scored at 14 DAI. For panels **(E,F)** the virulence and disease index were determined as in figure.

As *FolAsp* deletion reduced Fol virulence in tomatoes, it is believed that overexpressing this protein should promote more severe *Fusarium* wilt disease. To verify this hypothesis, we overexpressed FolAsp-GFP fusion protein [FolAsp (OE)] in *Fol* (Figure 3A and Supplementary Figure 1C). Phenotypically, *FolAsp* overexpression did not affect vegetative hypha growth on PDA, CM, and MM (Figures 3B, C). As expected, the conidia production of FolAsp (OE) was not affected compared with the WT (Figure 3D). These results further indicated that FolAsp was not involved in the growth development of *Fol*. Then, we performed the disease assay inoculated with WT- or FolAsp-overexpressing strain *FolAsp* (OE). As shown in Figures 3E, F, overexpression of FolAsp significantly caused more severe disease symptoms compared with the WT, which was consistent with the reduced pathogenicity by the *FolAsp* deletion mutant.

### FolAsp locates at the apoplast in plant cells

**FIGURE 4:**
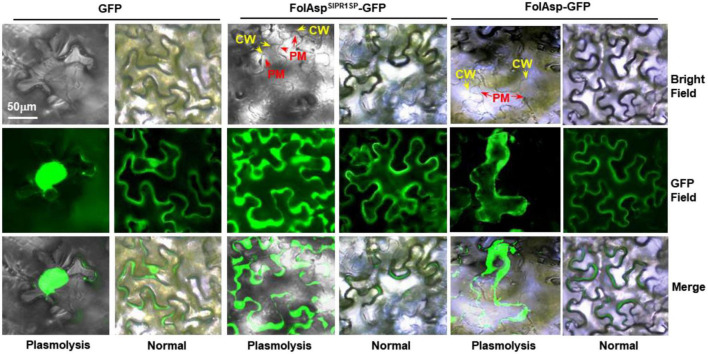
Subcellular localization of FolAsp in tobacco leaves. FolAsp-GFP indicates the fusion protein with its native N-terminal signal peptide. FolAsp^SlPR1SP^-GFP indicates replacement of its native signal peptide with the tomato PR1 signal peptide (N-terminal 1–24 amino acids). Images were taken at 3 DAI. The plasmolysis was visualized after treatment with 800 mM mannitol for 6 h. CM, cell wall; PM, plasma membrane.

To explore the localization and biological function of FolAsp, we transiently expressed FolAsp-GFP fusion proteins, in which green fluorescent protein (GFP) was added to the C-terminus of FolAsp protein. *Nicotiana benthamiana* leaves were used to determine subcellular localization using *Agrobacterium tumefaciens* infiltration by observing the GFP signal at 48 h. To increase protein levels for visualization, these constructs were expressed by the 35S promoter. Using a fluorescence microscope, FolAsp-GFP was detected in the plant cytoplasm with a stronger fluorescent signal (Figure 4). As FolAsp was identified to be secreted, it might function in the apoplast. Though FolAsp was transiently expressed with its native N-terminal signal peptide, FolAsp might not be faithfully secreted *in planta*, as described previously (Li et al., 2022).

The signal peptide of PR1 is highly effective in targeting proteins/peptides to the apoplast *via* the ER/Golgi secretory pathway (Kloppholz et al., 2011). To manipulate FolAsp secretion in tobacco, we replaced its native signal peptide with that of tomato SlPR1 (Li et al., 2022), named FolAsp^SlPR1*SP*^-GFP, and found that FolAsp^SlPR1*SP*^-GFP location was dramatically changed, but it was difficult to distinguish between apoplast and cytoplasm. Therefore, we adopted the plasmolysis assay. Following treatment with 800 mM mannitol for 6 h, plasmolysis was greatly observed, with a clear observation of the FolAsp^SlPR1SP^ protein in the apoplast. However, both the FolAsp-GFP and GFP were located in the cytoplasm (Figure 4). These results indicated that FolAsp might locate at apoplast *in planta* after secreting from *Fol*.

### FolAsp inhibits plant cell death (PCD)

**FIGURE 5:**
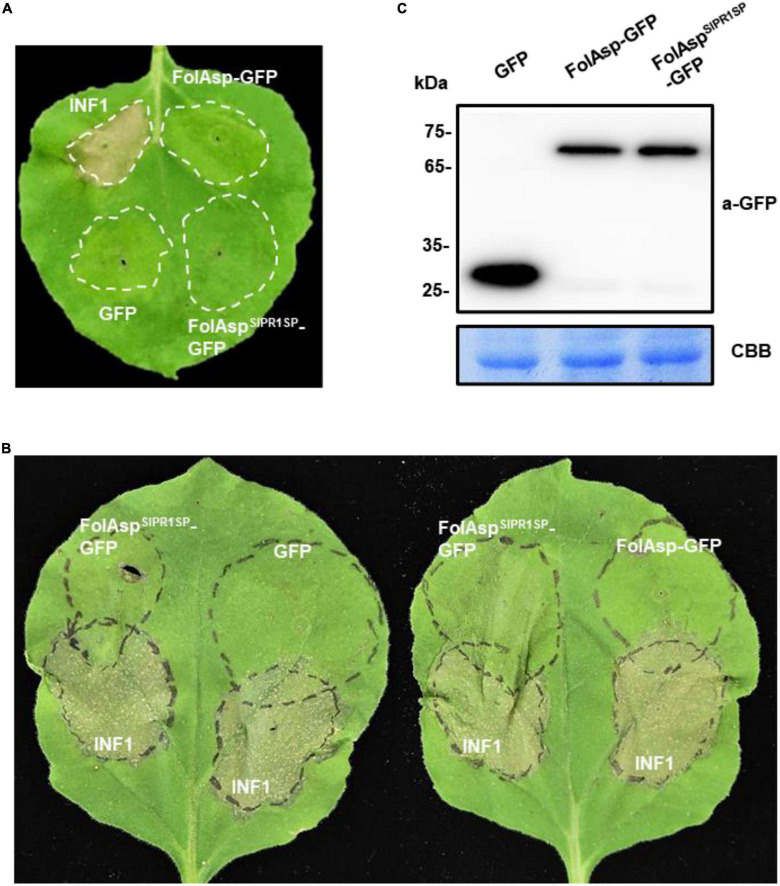
FolAsp inhibits INF1-mediated cell death. **(A)** Validation of FolAsp in induced cell death in *N. benthamiana.* Leaves were with agroinfiltration expressing GFP, FolAsp-GFP, and FolAsp^SlPR1*SP*^-GFP, or the cell death inducer INF1. Photographs were taken 3 DAI. **(B)** FolAsp is involved in suppression of cell death. Representative leaves showed cell death induced by transient expression of INF1 in *N. benthamiana* leaves pretreated with agroinfiltration expressing GFP, FolAsp-GFP, or FolAsp^SlPR1*SP*^-GFP for 2 days. The INF1-infiltrated leaves were photographed at 60 HAI. **(C)** Protein expression at 2 DAI prior to INF1 infiltration was monitored by Western blotting with anti-GFP antibody. Protein loading is indicated by CBB staining. Experiments were repeated at least three times with similar results.

To elucidate whether the transient expression of FolAsp alters plant immune responses, the local hypersensitive response (HR) was analyzed in *N. benthamiana*-using *A. tumefaciens*-mediated transient expression assay. The FolAsp-GFP protein was infiltrated into the leaves of tobacco through the stoma. A secreted FolAsp (FolAsp^SlPR1*SP*^-GFP) was also expressed to determine whether apoplastic localization works for the induction of plant immune responses. After 3 days of infiltration, there was no visible cell death detected in GFP-expressing control, FolAsp-GFP, and FolAsp^SlPR1*SP*^-GFP-expressing plants on the tobacco leaves (Figure 5A). By contrast, the transient expression of a pathogen-associated molecular pattern (PAMP) protein INF1 triggered significant PCD at the infiltration site, suggesting that the transient expression of FolAsp does not trigger the plant HR activity in tobacco. The mature protein was also determined by Western blotting analysis using an anti-GFP antibody (Figure 5B).

To examine whether FolAsp acts as an effector to inhibit defense responses during host–pathogen interactions, we determined the effector virulence function in suppressing PCD induced by INF1 in *N. benthamiana*. Strikingly, the sites infiltrated with FolAsp^SlPR1*SP*^-GFP 2 days and then challenged with the bacteria suspension carrying INF1 exhibited inhibited PCD 60 h later at the overlapped infiltration area, compared with agroinfiltration of GFP or FolAsp-GFP prior to INF1 incubation (Figure 5B). The mature protein was determined by Western blotting analysis using an anti-GFP antibody (Figure 5C). These results suggested that apoplast FolAsp (FolAsp^SlPR1*SP*^-GFP) suppresses PCD induced by INF1 and alludes to the possible virulence function of FolAsp, resulting in enhanced susceptible to the fungal pathogen *Fol*.

### FolAsp inhibits plant ROS production

**FIGURE 6:**
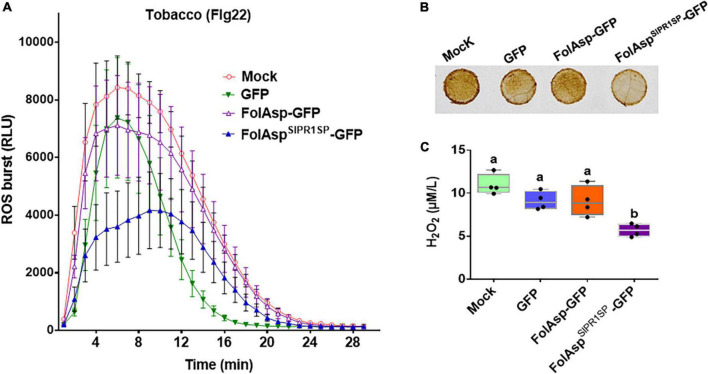
FolAsp inhibits ROS production. **(A)** Burst of ROS in GFP, FolAsp-GFP, or FolAsp^SlPR1*SP*^-GFP transiently expressed tobacco leaves was measured with 50 nM flg22. Mean values (±SEM) of eight replicates are shown. **(B)** FolAsp inhibits leaf H_2_O_2_ production induced by flg22. The GFP, FolAsp-GFP, or FolAsp^SlPR1*SP*^-GFP transiently expressed tobacco leaves and the control leaves (Mock) were treated with the DAB following 50 nM flg22 treatment for 30 min to visualize H_2_O_2_ accumulation. **(C)** Quantification of H_2_O_2_ was measured with samples from **(B)**. Statistical significance was revealed by one-way ANOVA (mean ± SE of 3 independent biological replicates, *p* < 0.05). Experiments were repeated three times with similar results.

To further expand the bioactivity of FolAsp in response to plant immunity, we determined reactive oxygen species (ROS) levels in tobacco leaves transiently expressing GFP, FolAsp-GFP, and FolAsp^SlPR1*SP*^-GFP or of the control leaves (Mock). First, we determined whether FolAsp^SlPR1*SP*^-GFP inhibits PAMP-triggered immunity (PTI) in tobacco. In this instance, the bacterial flg22 was used, since it has been widely shown to induce ROS production as the MAMP (Ngou et al., 2022). In tobacco leaves treated with the flg22, ROS burst was significantly observed in the GFP-, FolAsp- GFP-, or FolAsp^SlPR1*SP*^-GFP-expressing leaves compared with the control leaves (Figure 6A). However, the ROS burst was markedly suppressed in the FolAsp^SlPR1*SP*^-GFP-expressed leaves compared with GFP- or FolAsp-GFP-expressed leaves. Furthermore, the effector-mediated ROS inhibition was determined by DAB staining and H_2_O_2_ quantification. As can be seen in Figures 6B, C, flg22-mediated ROS accumulation in tobacco could be significantly suppressed with expressing FolAsp^SlPR1*SP*^-GFP; however, it is not altered on the GFP- or FolAsp-GFP-expressing leaves, compared with the control leaves (Mock). This result indicated that apoplast FolAsp^SlPR1*SP*^-GFP effectively decreased H_2_O_2_ production. This suggested that FolAsp was likely an important apoplast protein to inhibit MAMP-induced basal immunity.

### FolAsp possesses protease activity

**FIGURE 7:**
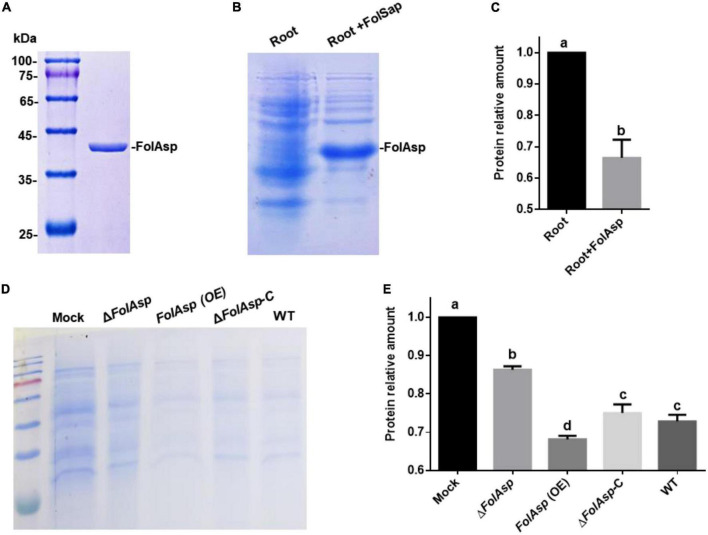
FolAsp possesses protease activity on host protein. **(A)** CBB-stained SDS-PAGE gel of purified FolAsp-his protein is shown. Heterologous expression of recombinant FolAsp-his protein using *Escherichia coli* BL21. Extracted protein was incubated with Ni-NTA agarose and then eluted with imidazole. **(B)** FolSap promoted degradation of host protein. The extracted root protein was inoculated with 1 μg purified FolSap for 2 h. CBB-stained SDS-PAGE gel of supernatant fractions is shown. **(C)** Quantification of the relative root protein abundance after FolAsp treatment, as in **(B)**. **(D)** Amount of tomato protein following treatment with the indicated strains or without any treatment as the control (Mock). The root protein was extracted and incubated with the spores of the indicated strains for 4 h. CBB-stained SDS-PAGE gel of supernatant fractions is shown. **(E)** Quantification of root proteins treated with *Fol* strains relative to Mock, as in **(D)**. For **(C,E)**, statistical significance was revealed by one-way ANOVA (mean ± SE of 3 independent biological replicates, *p* < 0.05).

The predicted aspartic protease domain indicated FolAsp might possess protease activity. To test this hypothesis, we expressed the protein in *Escherichia coli* BL21 (DE3) to purify it for enzyme activity analysis. The FolAsp protein was expressed in a supernatant culture and used for protein purification. The results of Coomassie brilliant blue staining revealed the obtained FolAsp protein (Figure 7A). Subsequently, root protein was used as the substrate to detect the protease activity of FolAsp *in vitro*. As can be seen in Figures 7B, C, adding FolAsp could remarkably promote host protein degradation. The results revealed that the FolAsp possessed protease activity on host protein.

Furthermore, we detected the *Fol* transformants promoted protein degradation on host protein to evaluate the contribution of FolSap on the protease activity. Following incubation with extracted root protein as the substrate, the results of Coomassie brilliant blue staining revealed that all *Fol* strains could promote root protein degradation compared with the control (Mock) (Figures 7D, E). However, FolAsp overexpression (Supplementary Figure 1A) significantly increased protein degradation compared with the WT and Δ*FolAsp*-C strains. Conversely, FolAsp deletion remarkably decreased protein degradation. These results indicated that FolAsp can degrade host protein through its protease activity.

Taken together, these results suggested that secreted protease FolAsp might inhibit plant basal defense or possess host protein degradation in apoplast space to facilitate its virulence on the host.

## Discussion

Most effectors identified from *Fol* were originally identified in the xylem sap of *Fol*-infected tomato plants, such as secreted in xylem (Six) proteins, which are important for pathogenicity (Houterman et al., 2007; Gawehns et al., 2015). However, the identification of secreted proteins beyond the six proteins is poorly understood in *Fol*, especially during the initial infection stages. We previously performed a secretome-wide analysis in *Fol* to provide new sight of infection-related proteins and found that most of them with an N-terminal SP were annotated as enzymes (Li J. et al., 2020). To date, three secreted proteins are shown to contribute virulence, and two of them encode the ribonuclease and peptidase, respectively (Li et al., 2022; Qian et al., 2022a,b). In this study, we characterized one aspartic protease FolAsp from the *Fol* secretome. Deletion of the *FolAsp* significantly decreases its pathogenicity to host tomato, indicating that secreted FolAsp is involved in the virulence of pathogen *Fol*.

Secreted proteins/effectors play important roles in the invading pathogen by blocking plant immunity or altering specific plant processes (Zhang et al., 2017; Cao et al., 2018). The N-terminal signal peptide of FolAsp displayed secretory activity in the yeast system, indicating that FolAsp is possibly secreted *via* the ER/Golgi secretory pathway (Kloppholz et al., 2011). In the biotrophic fungus *Fol*, whose growth is confined to the apoplast space, the signal peptide sequences might dominate the secretion and location of FolAsp in the apoplastic space of the host. To support it, the transiently expressed FolAsp with a plant signal peptide revealed its location at apoplastic space *in planta*. Therefore, FolAsp probably functions at the plant apoplast during *Fol* infection.

Proteases have been described as playing an important role in the host invasion process in many pathogenic microorganisms. Extracellular proteases from pathogenic fungi fulfill a number of specialized functions during the infective process in addition to the simple role of digesting molecules for nutrient acquisition (de Assis Tacco et al., 2009). Some studies investigating the role of extracellular aspartyl proteases in pathogenesis have focused on fungi (Li C. et al., 2020). In *A. fumigatus*, the aspergillopepsin aspartic protease is secreted in large amounts during infection of the mouse lung, facilitating fungus penetration (Lee and Kolattukudy, 1995). The FolAsp, identified from root-induced secretome (Li J. et al., 2020), is specifically upregulated induced with host root. Similarly, two ASP domain-containing proteases were also upregulated during infection of *Sclerotinia sclerotiorum*. An aspartyl (acid) protease, Sscle04g035550, was upregulated at all time points commonly associated with plant pathogenic protease activity (de Assis Tacco et al., 2009). Another aspartate protease, Sscle07g058540, was the second most upregulated protease at 24 hpi and shows homology to several aspergillopepsin-like proteins whose activity is important in invasive aspergillosis of humans (Rementeria et al., 2005). In *Monilinia laxa*, which is responsible for brown rots disease of stone and pome fruits, a secreted pepsin-like protein with a conserved domain for aspergillopepsin-like aspartic proteases was significantly upregulated by transcriptome analyses (De Miccolis Angelini et al., 2018). There results implied its important function in initial pathogenesis. As expected, deletion of *FolAsp* decreased its virulence in tomatoes; however, its growth phenotype was not affected. Furthermore, overexpression of FolAsp significantly promoted invasion and enhanced tomato susceptibility, suggesting that FolAsp may suppress the defense response of host plants upon *Fol* infection.

The identified FolAsp (FOXG_03994) had widely conserved homologs in fungi, indicating that this protein might be involved in general pathogenicity by conserved mechanisms. The degradative properties of secreted proteases have attracted much attention as potential mediators of fungal invasion in infected tissue (Naglik et al., 2003). Numerous functions have been attributed to aspartyl proteases in microorganisms, which range from nutrient degradation to the activation of signaling molecules (de Assis Tacco et al., 2009). In addition, aspartyl proteases from fungi serve to activate other zymogens such as alkaline phosphatase, chitin synthase, and other proteases. Aspartyl proteases from *Schistosoma* species are known to be responsible for host-specific proteolytic degradation of mammalian hemoglobin (Koehler et al., 2007). In this regard, the fact that the host protein is less abundant treated with FolAsp protein or FolAsp overexpression strain would point to its importance of enzymatic activity in the pathogenesis of the organism. The biochemical functions of many Avr proteins identified from various plant pathogens mainly constitute enzymatic activities (Song et al., 2020). However, the biochemical role of the aspartyl protease FolAsp in *Fol* still remains unclear. Future work will focus on its biochemical characterization.

When pathogens infect and secrete effectors to host cells, the basal defense responses of plants, such as ROS bursts, resistance gene expression, and cell death, are activated to inhibit the pathogen growth and development (Lyu et al., 2016; Kettles et al., 2018; Yang et al., 2018). In our study, transient expression of *FolAsp* protein in *N. benthamiana* did not induce cell death, similar to the recent findings of a peptidase effector FoAPY1 from *Fol*, which does not have the ability to induce cell death (Qian et al., 2022b). This family of aspartate proteases is also annotated by the MEROPS database as the peptidase family A1 (pepsin A, clan AA) (Santos et al., 2013). In addition to degrading host proteins into amino acids for nutrient acquisition or catabolic activities (Lowe et al., 2015), peptidases are reported to possess other functions in host plant–pathogen interactions. HopN1, a secreted protein from the bacterium *Pseudomonas syringae*, is a cysteine peptidase that cleaves the host PsbQ protein, which is a key photosynthesis enzyme, to block programmed cell death (Rodríguez-Herva et al., 2012). Similarly, the FolAsp could inhibit INF1, a MAMP identified from *Phytophthora* species, and induce cell death (Qiu et al., 2022). Further study revealed that FolAsp could suppress flg22-activated ROS production and H_2_O_2_ accumulation. Plants produce ROS to restrict pathogen invasion. A recent report revealed that ROS detoxification employed by *Botrytis cinerea* facilitates fungal invasion (Yang et al., 2022). The results indicated that the FolAsp protein might not be similar to other Avr effectors in terms of its cytotoxic effects in the plant or may not be recognized in the host cell. Instead, FolAsp likely suppresses the defense response of host plants by arresting ROS production to promote pathogen invasion.

## Conclusion

The results showed that FolAsp significantly promoted invasion and enhanced host susceptibility to fungal pathogens, suggesting that FolAsp may suppress the defense response of host plants during *F. oxysporum* infection. These findings provide insight into the interactions between *F. oxysporum* and host plants, contributing to the understanding of the pathogenic mechanism of this fungus. With the extensive study on the structure and function of secreted aspartic protease, this kind of virulence factor was supposed to be a potential target for the development of novel antifungal agents.

## Data availability statement

The original contributions presented in this study are included in the article/Supplementary material, further inquiries can be directed to the corresponding authors.

## Author contributions

CW, YZ, ZL, and YQ carried out the experiments. YL, LY, and SL performed technical supports and statistical analysis. WL and JL initiated the project, designed the experiments, analyzed the data, and provided funding. JL, CW, and WL wrote the manuscript with inputs from all the authors. All authors contributed to the article and approved the submitted version.

## Footnotes

1
http://www.cbs.dtu.dk/services/SignalP/
