# Supplementary material for: The secreted FolAsp aspartic protease facilitates the virulence of Fusarium oxysporum f. sp. lycopersici
Source: Front Microbiol. 2023 Jan 25;14:1103418. doi: 10.3389/fmicb.2023.1103418 (PMC9905682; doi:10.3389/fmicb.2023.1103418)
Supplement: Supplementary file 1 [file Data_Sheet_1.docx]

Supplementary figure legends


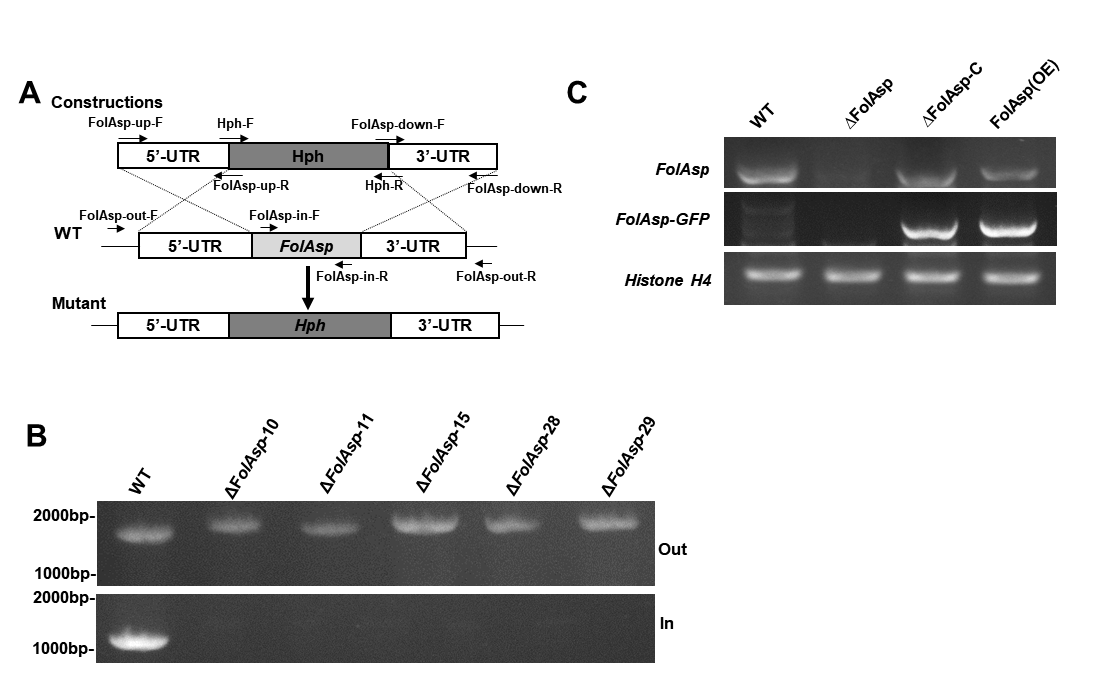


**Figure S1.** Generation of *FolAsp* mutant strains. (A) Homologous recombination-based deletion of *FolAsp*. The primers used for gene deletion and PCR identification was indicated in the diagram. (B) PCR analysis to identify the ∆*FolAsp* (KO10, KO11, KO15, KO28 and KO29) with primers FolAsp-out-F/R and FolAsp-in-F/R as shown in (A). (C) PCR analysis to determine the complementary strain ∆*FolAsp*-C under its native promoter and *FolAsp* overexpression (OE) strain under the PR27 constitutive promoter. Primer pair FolAsp-PQB-F/R for amplification across the FolAsp coding sequence. The *FolAsp* forward primer FolAsp-PQB-F and the vector reverse primer pYF11-cGFP-R located at C-terminal GFP were used for amplification of the FolAsp fused GFP coding sequences. Primer pair H4-qRT-F/R specific to Histone H4 (FOXG_09402) was used as a positive PCR control.


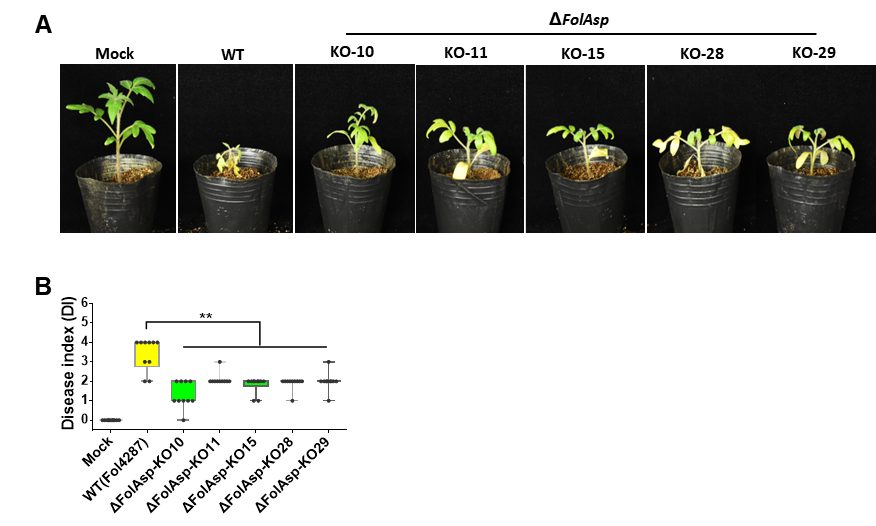


**Figure S2**. Deleting *FolAsp* attenuated the pathogenicity on hosts. (A) Virulence of the WT and *FolAsp* knock-out mutant strains on tomato. The disease symptoms were observed, and photographs were taken at 14 days after inoculation (DAI). Mock, inoculation with water. (B) Disease index scored at 14 DAI. Star represents significant differences according to one-way ANOVA (***P*< 0.01, n=9). Experiments were repeated three times with similar results.
