# Supplementary material for: The secreted FolAsp aspartic protease facilitates the virulence of Fusarium oxysporum f. sp. lycopersici
Source: Front Microbiol. 2023 Jan 25;14:1103418. doi: 10.3389/fmicb.2023.1103418 (PMC9905682; doi:10.3389/fmicb.2023.1103418)
Supplement: Supplementary file 2 [file Table_1.DOCX]

Table S1. Primers used in this research

| Primer name | Sequence |
| --- | --- |
| Hph-F | 5’-GGCTTGGCTGGAGCTAGTGGAGGTC-3’ |
| Hph-R | 5’-AACCCGCGGTCGGCATCTACTCTAT-3’ |
| FolAsp-up-F | 5’-CCGCCGTCTCAACCAATAGC-3’ |
| FolAsp-up-R | 5’-GACCTCCACTAGCTCCAGCCAAGCC GAGATAGAGATAAGACCAGA-3’ |
| FolAsp-down-F | 5’-ATAGAGTAGATGCCGACCGCGGGTT AGTCTGTAAATAGCCGGATC-3’ |
| FolAsp-down-R | 5’-ATGGCACATCAGAATTGAAG-3’ |
| FolAsp-out-F | 5’-TTGATGGAAGCAGCCAAGAA-3’ |
| FolAsp-out-R | 5’-TTCACATGGTTTCGCATGAT-3’ |
| FolAsp-in-F | 5’-TCTCTGGCTTTTGCTTCCAT-3’ |
| FolAsp-in-R | 5’-GCAATATCACCGAAGATGTTG-3’ |
| FolAsp-PQB-F | 5’-ATGCCTTCCATCAACGCTCT-3’ |
| FolAsp-PQB-R | 5’-CAGTGTCTTCTTGGCCCAGC-3’ |
| FolAspSlPR1SP-PQB-F | 5’-ATGGGGTTGTTCAACATCTCATTGTTACTCACTTGTCTCATGGTATTAGCC  ATATTTCACTCTTGTGAGGCCGCCCCTGCTGCCCAGGACAAG-3’ |
| pYF11-cGFP-R | 5’-CTTGTACAGCTCGTCCATGC-3’ |
| FolAsp(OE)-F | 5’-TTTCGTAGGAACCCAATCTTCAAAATGCCTTCCATCAACGCTCTTCTCAC  AGCCTC-3’ |
| FolAsp-C-F | 5’-ACTCACTATAGGGCGAATTGGGTACTCAAATTGGTTGCACTTGTCTCAGT  TTAGC-3’ |
| FolAsp-C-R | 5’-CACCACCCCGGTGAACAGCTCCTCGCCCTTGCTCACCAGTGTCTTCTTGG  CCCAGC-3’ |
| pSUC2-F | 5’-AATTCATGCCTTCCATCAACGCTCTTCTCACAGCCTCTCTGGCTTTTGCTT  CCATCGCCCTCGGCC-3’ |
| pSUC2-R | 5’-TCGAGGCCGAGGGCGATGGAAGCAAAAGCCAGAGAGGCTGTGAGAAGA  GCGTTGATGGAAGGCATG-3’ |
| pET-28a-F | 5’-CTGGTGCCGCGCGGCAGCCATATGCCTTCCATCAACGCTCT-3’ |
| pET-28a-R | 5’-CACCAGTCATGCTAGCCATCAGTGTCTTCTTGGCCCAGC-3’ |
| FolAsp-qRT-F | 5’-TCTGACGAGGATGTCGAGTG-3’ |
| FolAsp-qRT-R | 5’-CTTAGCTGTGCTGCTCTTGG-3’ |
| H4-qRT-F | 5’-CCAAGCGTCACCGAAAGATT-3’ |
| H4-qRT-R | 5’-CCCTCGAGGAAGGTCTTGAG-3’ |
